# Polydeoxyribonucleotide (PDRN) in Post-procedure Recovery in Aesthetic Medicine: A Narrative Review

**DOI:** 10.7759/cureus.108886

**Published:** 2026-05-15

**Authors:** Julio César Flores Rodríguez, Luiz Eduardo Toledo Avelar, Kyuho Yi, Rodrigo Merino Arellano, Brenda Mariel Porras Zamora, Lorena Valdovinos Martínez

**Affiliations:** 1 Aesthetic and Regenerative Medicine, Clinica Aura Medicina Estética, Monterrey, MEX; 2 Aesthetic and Regenerative Medicine, Sociedad Mexicana de Investigación en Medicina Estética (SOMIME), Monterrey, MEX; 3 Aesthetic and Regenerative Medicine, Sociedade Brasileira de Cirurgia Plástica (SBCP), Belo Horizonte, BRA; 4 Anatomy, Yonsei University, Seoul, KOR; 5 Plastic Surgery, TecSalud, Monterrey, MEX; 6 Aesthetic and Regenerative Medicine, Consultorio Particular Dra. Brenda Mariel Porras Zamora, Mexico City, MEX; 7 Aesthetic and Regenerative Medicine, Sociedad Mexicana de Investigación en Medicina Estética (SOMIME), Mexico City, MEX; 8 Aesthetic and Regenerative Medicine, Consultorio Particular Dra.Lorena Valdovinos Martínez, Zapopan, MEX; 9 Aesthetic and Regenerative Medicine, Sociedad Mexicana de Investigación en Medicina Estética (SOMIME), Zapopan, MEX

**Keywords:** adenosine a2a receptor, aesthetic medicine clinic, chemical peel, laser skin resurfacing, polydeoxyribonucleotide, polynucleotide, post-procedure recovery, skin erythema, skin regeneration, ­wound healing

## Abstract

Polydeoxyribonucleotide (PDRN) has been shown to have consistent regenerative and anti-inflammatory properties in a wide range of applications. Selective activation of the A2A receptor (A2AR) and induction of the phosphate scavenger system constitute its primary actions, which overlap the mechanism of skin regeneration after a surgical procedure; thus, PDRN can potentially be added to the post-surgical aesthetic surgery protocol of laser resurfacing, chemical peeling, microneedling, and radiofrequency. Literature search was conducted in the PubMed/MEDLINE (Medical Literature Analysis and Retrieval System Online), Scopus, and Cochrane Central Register of Controlled Trials (CENTRAL) databases with the following keywords: polydeoxyribonucleotide (PDRN) or polynucleotide, wound repair, aesthetic recovery, erythema, scar, lasers, and skin regeneration. No stipulated date limit was applied. Interventional and observational human studies, any available relevant preclinical evidence, and published systematic reviews were included. The studies were chosen based on their applicability to the biological processes of PDRN and their clinical application in skin repair situations that can be implemented in aesthetic practice. Narrative synthesis was used for thematic analysis. The pro-resolving immune reaction of PDRN works through a two-pronged mechanism: balancing the pro-inflammatory cytokine A2AR-mediated action (tumor necrosis factor-alpha (TNF-α), interleukin (IL)-6, and IL-1 beta) and vascular endothelial growth factor-mediated nucleotide provisioning to growing keratinocytes and fibroblasts. There is human clinical evidence (including randomized controlled trials (RCTs), comparative cohort studies, and split-face trials) that PDRN enhances the period of re-epithelialization, erythema, scarring, and melanogenesis, which are the direct clinical outcomes with respect to post-procedural aesthetic recovery. PDRN has also shown non-inferior short-term cosmetic results compared to hyaluronic acid filler, with indications of greater biostimulatory stability. The adverse event profile in all published studies is invariably positive. The limitations of the technique, however, are a lack of RCTs (with human aesthetic cohorts), small sample sizes, variability in formulations, and a lack of long-term follow-up. Thus, the findings discussed in the current paper make PDRN an appealing and bio-plausible clinical partner in enhancing post-surgical recovery of patients undergoing aesthetic surgery. The available mechanistic and translational evidence establishes a credible biological basis for integrating PDRN into post-procedural care. On the basis of this evidence, its use may be considered a reasonable adjunct in clinical practice, subject to individual patient assessment and institutional protocol.

## Introduction and background

Skin rejuvenation/repair has become a booming market over the last 20 years; ablative and non-ablative laser resurfacing, chemical peels, microneedling (conceptualized with or without radiofrequency), and injectable biostimulators are now considered the primary modes of clinical practice in aesthetic dermatology across the globe [1,2]. These modalities operate through a regulated amount of tissue injury, stimulating the intrinsic wound healing cascade in the skin to achieve clinical endpoints such as a decrease in wrinkle depth, enhanced texture, repair of pigment, and dermal collagen remodeling [3]. The post-procedural recovery phase, which includes erythema, temporary epidermal barrier damage, edema, crusting, and re-epithelialization, is thus not a negative effect that needs to be endured but rather a physiological event whose optimization directly determines the safety, efficacy, and patient tolerability of the procedure.

The healing is of considerable clinical significance. Delays in healing of late erythema after a fractional CO₂ laser resurfacing could take weeks or months for chronically ill patients to recover, and this prolonged recovery is also the top cause of patient dissatisfaction with the procedure and deterrence of second or subsequent cosmetic surgeries [4,5]. Post-inflammatory hyperpigmentation (PIH) is a side effect that happens in Fitzpatrick skin phototype types III-VI at disproportionately high rates, particularly in patients with darker skin after experiencing aggressive resurfacing [6,7]. It has been known that ablative procedures lead to the emergence of hypertrophic scarring, particularly when control is lost during inflammation of the post-procedural period [8,9]. Though the clinical significance of such postoperative complications in the recovery period is greatly recognized, pharmacological intervention in hastening and maximizing post-procedural healing is not as firmly established as the technologies used during the procedure, which has made the latest post-procedural protocols, such as emollients, broad-spectrum sunscreens, topical corticosteroids, and, more recently, platelet-rich plasma (PRP) and growth factor stocks, to be more or less empirically driven and eclectic [10,11].

Polydeoxyribonucleotide (PDRN) has become a competitor because it has a strong biological rationale and a growing but still immature foundation for post-surgical aesthetic recovery that can be leveraged for this purpose. PDRN is a high-molecular-weight (50-1,500 kDa) purified deoxyribonucleotide polymer that is produced from sperm of *Oncorhynchus mykiss* (rainbow trout) or *Oncorhynchus keta* (chum salmon). It is prepared by controlled elimination processes, deproteinization, and sterilization to eliminate any trace of proteins and peptides that might cause immunogenic reactions and to fabricate a biologically competent non-immunogenic polymer [12]. PDRN was initially approved by the Italian Medicines Agency (AIFA) in 1994 as a therapy for skin ulcers and dystrophic connective tissue disorders, as well as superficial wounds, and has a history of more than 30 years of real-world utility in clinical practice [13]. More recently, PDRN-based medical devices such as dermal fillers and biostimulatory injectables have been approved by regulators in countries (e.g., South Korea or those in Europe) that increasingly acknowledge their use in aesthetics [14]. 

PDRN and polynucleotide (PN) are both DNA-derived biopolymers sourced from salmon sperm, yet differ fundamentally in molecular weight and mechanism. PDRN comprises shorter deoxyribonucleotide chains below 1,500 kDa, whereas PN denotes longer chains at or exceeding this threshold. PDRN acts primarily through adenosine A2A receptor activation and the nucleotide salvage pathway, driving anti-inflammatory, angiogenic, and tissue-repair responses. PN, by contrast, forms a highly viscous three-dimensional dermal matrix and exerts its effects predominantly through extracellular matrix remodeling. These distinctions carry direct clinical implications for treatment selection in regenerative and aesthetic dermatology [14-16].

The regenerative action of PDRN involves two mechanisms: (i) A2AR-mediated inhibition of pro-inflammatory cytokines (tumor necrosis factor-alpha (TNF-α), interleukin (IL)-2, and IL-1β) and up-regulation of the vascular endothelial growth factor (VEGF), and (ii) salvage-pathway-mediated provision of nucleotides to expanding keratinocytes and fibroblasts [15]. The human clinical data, in the form of randomized controlled trials (RCTs), comparative cohort studies, and split-face trials, consistently show that PDRN speeds up the re-epithelialization process, shortens the duration of erythema and scarring, and inhibits the process of melanogenesis, with results directly implying post-procedural aesthetic recovery [12,16].

This literature review aims to harmonize available mechanistic, preclinical, and clinical data regarding PDRN for recovery following aesthetic procedures. It is structured thematically, beginning with the biological effects underlying the regenerative potential of PDRN, then moving on to the procedural backgrounds most pertinent to the practice of aesthetics, and concluding with a reflection on the shortcomings and opportunities of the existing evidence and future directions for research. Hence, most of the current evidence is indirect and largely based on wound-healing and translational studies rather than direct aesthetic clinical trials.

Methodology

This is a narrative literature review. PubMed/MEDLINE (Medical Literature Analysis and Retrieval System Online), Scopus, and Cochrane Central Register of Controlled Trials (CENTRAL) electronic databases were used for the literature search. The following keywords and search terms, such as "polydeoxyribonucleotide", "PDRN", "polynucleotide", "wound healing", "aesthetic recovery", "erythema", "scar", "laser", and "skin regeneration" were used. There was no set date limit. The search string for each database is given in Table 1. 

**Table 1:** Search strings for literature search PDRN: polydeoxyribonucleotidel; tiab: title abstract; MeSH: Medical Subject Heading

Database	Search Strings
PubMed	("polydeoxyribonucleotide"[tiab] OR "PDRN"[tiab] OR "polynucleotide"[tiab] OR "polydeoxyribonucleotide"[MeSH Terms]) AND ("wound healing"[tiab] OR "wound healing"[MeSH Terms] OR "aesthetic recovery"[tiab] OR "skin regeneration"[tiab] OR "tissue regeneration"[MeSH Terms] OR "erythema"[tiab] OR "erythema"[MeSH Terms] OR "scar"[tiab] OR "cicatrix"[MeSH Terms] OR "keloid"[MeSH Terms] OR "laser"[tiab] OR "lasers"[MeSH Terms])
Scopus	TITLE-ABS-KEY ( ( "polydeoxyribonucleotide" OR "PDRN" OR "polynucleotide" ) AND ( "wound healing" OR "aesthetic recovery" OR "skin regeneration" OR "erythema" OR "scar" OR "cicatrix" OR "keloid" OR "laser" ) )
Cochrane Library	#1 polydeoxyribonucleotide OR PDRN OR polynucleotide #2 "wound healing" OR "aesthetic recovery" OR "skin regeneration" OR erythema OR scar OR cicatrix OR keloid OR laser #3 #1 AND #2

**Figure 1:**
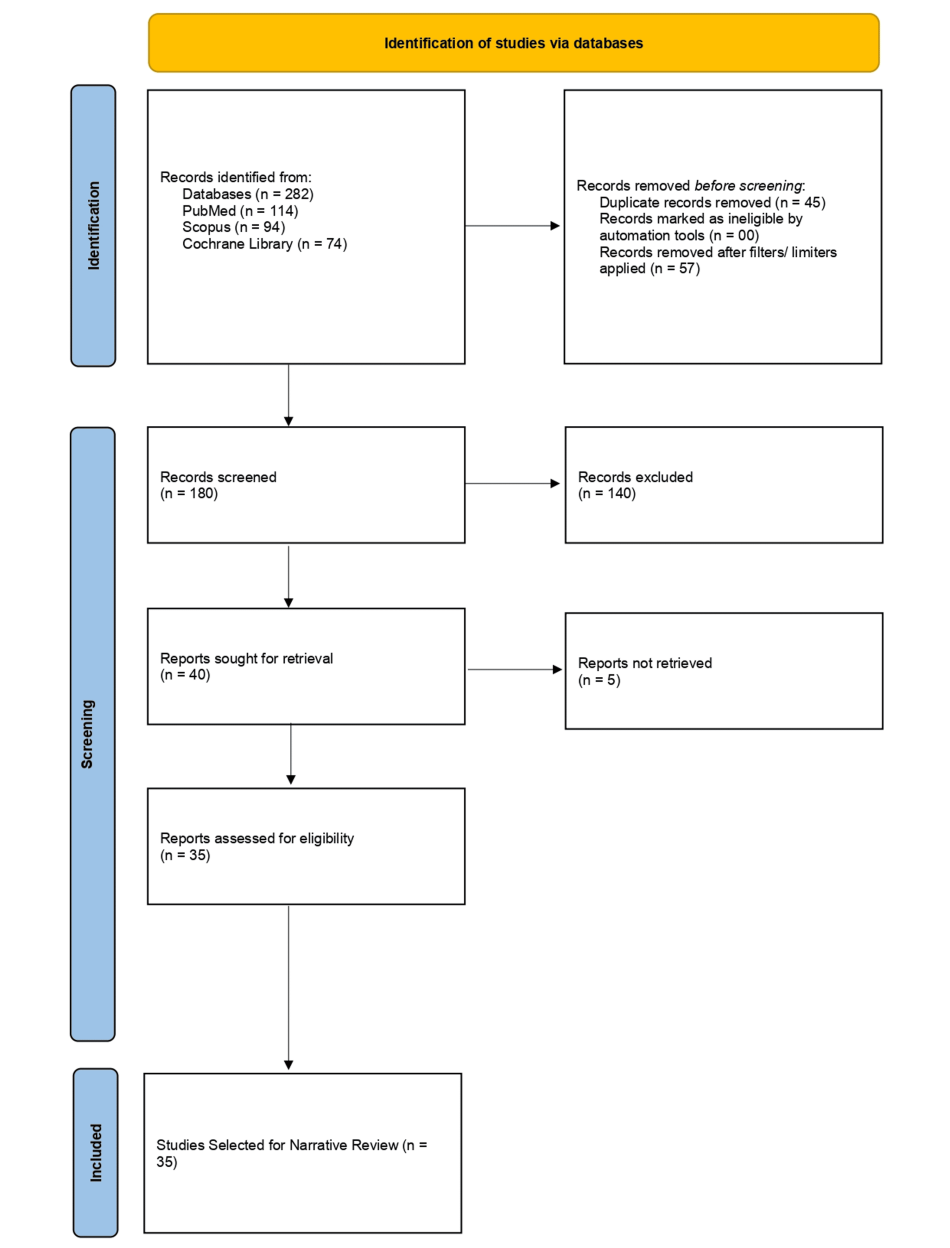
PRISMA flowchart showing selection process of studies PRISMA: Preferred Reporting Items for Systematic reviews and Meta-Analyses

Only English-language written articles were considered. The last date of the data search was April 16, 2026. The study selection process was performed according to the PRISMA 2020 guidelines checklist items, except risk of bias, statistical synthesis, certainty of evidence, and pooling of the quantitative data due to the narrative literature review study design (Figure 1). The included studies were both interventional and observational human studies, applicable preclinical evidence, and published systematic reviews. Studies were selected based on their relevance to the biological processes involved in PDRN and the practical use of PDRN in skin repair, which can aid aesthetic practice. Only English-language and peer-reviewed articles were included. The study is a narrative review, which synthesizes evidence using a thematic narrative approach.

## Review

Biological mechanisms of PDRN relevant to post-procedural recovery

Adenosine A2A Receptor Pathway

PDRN is a selective agonist of A2AR, a type of G protein-coupled receptor found on human skin fibroblasts, keratinocytes, endothelial cells, mast cells, and immune effector cells [12,17]. The A2AR-activating ligand stimulates a stimulatory G protein (Gs) and increases adenylate cyclase and cyclic adenosine monophosphate (cAMP). This increases cAMP, activates protein kinase A (PKA), and results in changes in the transcription factors nuclear factor kappa-light-chain-enhancer of activated B cells (NF-κB), cAMP response element binding protein (CREB), and hypoxia-induced factor 1 alpha [18]. This leads to the downregulation of the cytokines TNF-α, IL-6, and IL-1; the induction of VEGF expression; the promotion of fibroblast migration and proliferation; and the promotion of an organized extracellular matrix (ECM) deposition [12,19].

This means diminishing the inflammatory phase of skin lesions, which is the major source of tissue swelling, redness, and PIH, and accelerating the healing in the proliferative and remodeling phases post-procedure [20]. PDRN selectivity to A2AR activation, in comparison to non-selective adenosine receptor activation by adenosine or dipyridamole, has clinical consequences: non-selectivity is accompanied by cardiovascular and hemodynamic side effects limiting medication use, while selective agonism of A2AR is limited to anti-inflammatory and angiogenic effects in tissues [12].

Nucleotide Salvage Pathway

Concurrently, PDRN provides exogenous deoxyribonucleotides, which can be incorporated into the nucleotide miniaturization pathway, a resource-efficient metabolic pathway by which cells reuse nucleoside and nucleotide precursors in DNA synthesis without having to pass through the more costly de novo synthesis pathway [12], in association with A2AR. It is an autonomous complementary process to A2AR agonism and is particularly pertinent to the post-procedural period when nucleotide substrates are urgently required by cells as they are fast-moving to create a wound bed and fast-proliferating fibroblasts in the dermis. PDRN potentially facilitates faster cellular proliferation by supplying these defined substrates, enabling a shorter metabolically intensive de novo nucleotide production without disrupting the cell membrane, which can ultimately shorten the re-epithelialization schedule [21]. This twofold action, involving receptor-mediated signaling and direct metabolic substrate provision, makes PDRN unique among simple A2AR agonists and potentially explains why its wound healing effects are clinically significant and exceed the extent of clinical benefit of receptor pharmacology alone.

Microvascular Support and Angiogenic Stimulation

Post-procedural repair requires adequate tissue perfusion, providing oxygen, nutrients, and growth factors to proliferating cells of the wound bed and facilitating the removal of cellular debris and inflammatory mediators [22]. PDRN is a potent inducer of VEGF via the A2AR-mediated increase in hypoxia-inducible factor 1-alpha (HIF-1-alpha) protein, as well as direct stimulation of endothelial cells, and encourages neovascularization of healing tissue [23]. In preclinical in vivo models of ischemic skin flaps, PDRN treatment increased blood flow measured by laser Doppler imaging, increased VEGF mRNA expression, and increased the number of CD31-positive microvessels-results that support the functional angiogenic capacity of the compound [24]. This angiogenic activity has been found to help maintain the microvascular bed beneath the healing epidermis in the post-procedural context and to accelerate re-epithelialization [25]. However, it is the VEGF upregulation driving re-epithelialization that can initially manifest as pro-inflammatory conditions post-procedure [26]. Clinical evidence clarifies the role of PDRN in normalizing vascular architecture in the medium term, rather than merely temporarily diminishing inflammatory processes [27].

Synthesis and Remodeling of the ECM

PDRN increases collagen type I generation by stimulating the fibroblast's downstream signaling through A2AR, transforming growth factor-beta 1 (TGF-beta1), and the activation of focal adhesion kinase (FAK) phosphorylation [19]. In the healed wound, the healing process would result in an orderly deposition of collagen fibers that run parallel to the skin surface, the arrangement typical of normal scar tissue maturation, in place of disordered, thick strands of collagen fibers typical of hypertrophic scar tissue. Various preclinical wound healing tests have verified that PDRN-treated wounds showed an increased thickness of the granulation tissue, increased collagen content, and improved biomechanical characteristics when compared to the vehicle-treated control [12,25]. The further development of PDRN is based on these collagen-stimulating properties. As an anti-aging biostimulator, injectable PN/PDRN-based formulations are employed to replace age-associated collagen losses regardless of the procedural setting.

Anti-Melanogenic Properties

**Figure 2:**
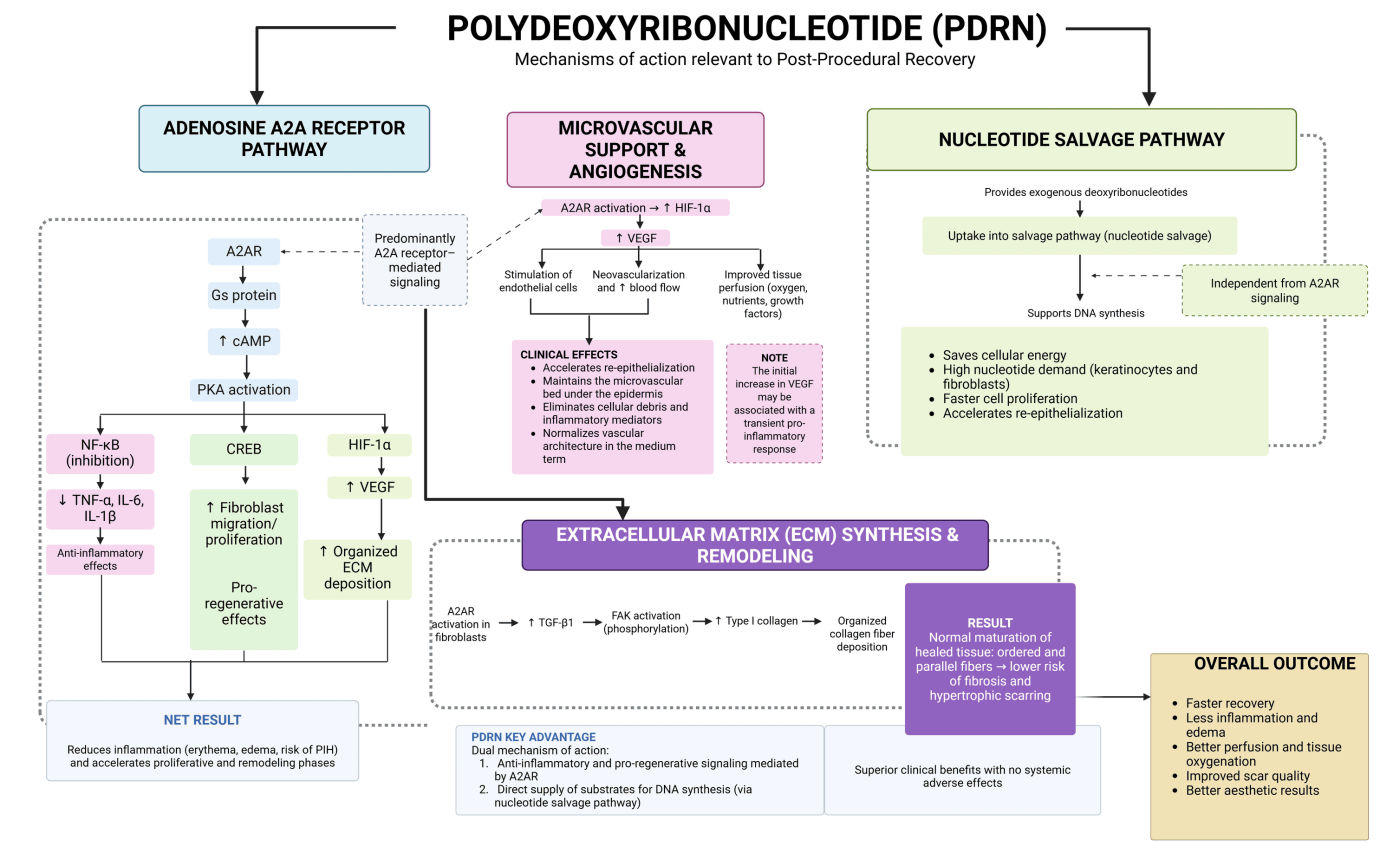
PDRN mechanism of action relevant to the post-procedure recovery PDRN: polydeoxyribonucleotide; A2Ar: adenosine A2A receptor; Stimulatory guanine  nucleotide-binding protein (Gs), Cyclic adenosine monophosphate (cAMP), Protein kinase A  (PKA), Nuclear factor kappa B (NF-KB), Tumor necrosis factor alpha (TNF-a), Interleukin 6  (IL-6), Interleukin 1 beta (IL-1ß). cAMP response element-binding protein (CREB), Hypoxia-  inducible factor 1 alpha (HIF-1a). Vascular endothelial growth factor (VEGF), Extracellular matrix (ECM), Transforming growth factor beta 1 (TGF-ß1), Focal adhesion kinase (FAK), Deoxyribonucleic acid (DNA), and Post-inflammatory hyperpigmentation (PIH). Image Credit: Julio César Flores Rodríguez; created in BioRender (https://biorender.com/isdtvfg)

One clinically important, yet not adequately recognized, action of PDRN is its ability to inhibit melanogenesis, which directly relates to PIH prevention after aesthetic surgeries [16]. An in vitro and pilot clinical study by Noh et al. indicated that PDRN inhibits the microphthalmia-related transcription factor (MITF), a master controller of melanocyte differentiation and melanin synthesis, as well as the rate-limiting enzymatic activities of tyrosinase and tyrosinase-related protein-1 (TRP-1) [16]. PDRN drastically decreased melanin content at concentrations in the range of 50-100 μg/mL (p < 0.05) in human melanocyte-keratinocyte co-cultures, which is the physiologically relevant cell system for studying PIH, and intracellular phosphorylation of ERK and AKT was elevated, illustrating signal transduction. The pilot clinical study involving six female patients with known cases of facial hyperpigmentation showed that all patients experienced at least a two-point reduction in a five-point clinical grading scale following three sessions of intradermal PDRN. Although this clinical study was not controlled and lacked sufficient power for statistical analysis, the mechanistic consistency of the in vitro and clinical results supports the biological plausibility of PDRN being a post-procedural PIH prophylaxis [16]. Figure 2 illustrates the PDRN mechanism of action relevant to the post-procedure recovery.

Clinical evidence for PDRN in skin repair and wound healing

Evidence from Surgical and Donor Site Wound Models

The closest analogy to ablative aesthetic procedure wounds is the controlled surgical wound, which involves disruption of tissue, either full-thickness or partial-thickness, in an immunocompetent host. This type of wound represents the most developed area of human clinical evidence on PDRN. A double-blind, placebo-controlled RCT that specifically investigates the effect of PDRN on re-epithelialization used 26 patients undergoing autologous skin grafting, randomizing them to receive either PDRN (5.625 mg intramuscularly daily plus subcutaneously every three days) or a placebo over a period of 10 days [2]. The PDRN group showed a statistically significantly higher percentage of re-epithelialization of donor sites than the placebo group at seven days (p < 0.008), and a higher and stronger trophic effect was observed over the period of observation. There were no reported adverse effects in either arm; therefore, this dosing regimen is safe in a controlled clinical setting.

Park et al. also conducted a study and found that PDRN improves angiogenesis, modifies cytokine profiles, and speeds tissue repair, improving chronic diabetic foot ulcers, pressure ulcers, and venous leg ulcers, as well as surgical donor site and corneal wound epithelial regeneration [28]. Cosmetic dermatology benefits from adjuvants. PDRN also has a low adverse event rate in studies and real-world applications. New delivery technologies and combination treatments have increased therapeutic possibilities. The findings integrate molecular insights with substantial clinical data to demonstrate PDRN's versatility and safety as a regenerative agent [28].

Building on this, Valdatta et al. conducted a pilot study comparing 40 patients, in which donor sites in the experimental group were covered with a PDRN-containing ointment and the control group with usual non-adherent gauze dressings [29]. The effect size was substantial; the mean time to complete re-epithelialization took 12.5 days in the PDRN group versus 24.45 days in controls (roughly 12 days in clinical terms, or almost a 50% reduction in healing time). Moreover, the changing of the dressing was consistently painless in the PDRN group, contrasting with the often painful procedures described in the controls. Furthermore, no wound infection was reported in the PDRN group compared to nine wound infections in the control group. Although the lack of either formal or internal unblinding, or the pilot study nature, presents a potential risk of performance bias, the reliability and clinical significance of both of these findings are persuasive. The reduction in pain during dressing changes can be directly applicable in the post-procedural aesthetic environment, where re-dressing, cleansing, and wound evaluation operations following ablative laser or deep chemical peel treatments are factors that produce extensive patient pain [29].

At the larger end of the evidence spectrum, Squadrito et al. have presented the findings of the largest placebo-controlled RCT in the PDRN wound healing literature, involving 216 participants with Wagner grades 1-2 chronic diabetic foot ulcers [30]. Participants received PDRN (5.625 mg) intramuscularly five days a week and perilesionally twice a week, or a placebo, for eight weeks. At week eight, the PDRN group had achieved full wound closure in about twice the percentage of patients compared to placebo, a major clinical finding. Although diabetic foot ulcers presented a significantly more challenging healing environment compared to immunocompromised individuals or patients undergoing aesthetic procedures, the sample size and methodological rigor of this trial established an upper limit for the extent of PDRN's wound healing activity, while also providing the smaller studies mentioned above with a stronger statistical basis for their limited sample sizes [30]. An RCT by Kim et al. with a similar design and 23 patients with pressure ulcers found that the wound surface area and Pressure Ulcer Scale for Healing (PUSH) scores decreased significantly over four weeks of treatment, with no adverse events observed [31]. Cumulatively, these studies on wound healing indicate a stable and reproducible biological signal: PDRN speeds tissue repair in various wound types and phases and in different patient groups when compared to placebo or usual care.

Laser and Energy-Based Procedure Model Evidence

The direct support for the utility of PDRN after aesthetic energy-based therapies is largely from procedural studies, stemming from a controlled preclinical trial of the fractional ablative CO₂ laser, a paradigmatic aesthetic rejuvenation device. A study using a rat model found that PDRN injection resulted in faster wound healing, significantly increased granulation tissue thickness, and vascular endothelial growth factor expression after fractional CO₂ laser treatment compared to a control group [32]. These results explicitly indicate that the PDRN wound healing mechanisms function in the post-laser wound setting and provide the biological basis of their use in post-laser recovery. This model, where a fractional ablative CO₂ laser forms discrete columns of thermal damage within spared tissue surrounding the wound, the same microscopic pattern seen in human fractional resurfacing, supports the hypothesis that the kinetics of wound healing in dorsal skin wounds in rats will find an equivalent in human wound healing biology.

Although ophthalmological, the corneal photorefractive keratectomy (PRK) model presents direct mechanistic evidence, as its excimer laser ablation of epithelial targets is a controlled and reproducible technique and is closely comparable to epidermal ablation in skin resurfacing. Lazzarotto et al. included 60 randomly selected eyes receiving PDRN eye drops or a placebo in their study, in which the PDRN group demonstrated a reduced mean disepithelialized corneal area on post-procedure day 2, showing a much faster recovery of the epithelium [33]. The participants tolerated the PDRN eye drops well. Although corneal epithelium and epidermal keratinocytes are different in several respects, they are both stratified epithelial tissues that heal by the same basic keratinocyte migration and proliferation pathways, and they both respond to A2AR stimulation. This study by Lazzarotto et al., thus, provides valuable proof-of-concept data showing that PDRN enhances the healing of laser-induced epithelial injury in a controlled, blinded human experiment, a finding that has not yet been reproduced in a skin resurfacing controlled clinical trial [33].

It has also been suggested in clinical practice that PN should be used following energy-based aesthetic procedures, a practice supported by both data from physician surveys and small observational series [14]. In a large survey of more than 235 Korean dermatologists and aesthetic physicians practicing aesthetic medicine, Rho et al. found that 71% of the survey participants were board-certified dermatologists with at least five years of expertise, and 88% of skin boosters used PN injection [34]. Fine cheek lines, infraorbital fine lines, periorbital fine lines, uneven skin texture, dry skin, and forehead fine wrinkles were the top six PN indicators. Many used a 33-G needle and repeated dermis puncture. PN injections are usually administered three times, four weeks apart. Of the PN users, 79% combined PN injection with lasers and light therapy, mostly radiofrequency (non-invasive, needle RF) and high-intensity focused ultrasound. Their survey found that Korean dermatologists used PN to enhance skin quality in their survey and found that facial fine lines were the best indicator of PN; therefore, it was typically repeated and supplemented with other non-surgical rejuvenation techniques [34].

Evidence from Scar Prevention and Post-Surgical Wound Management

Kim et al. conducted the most rigorously designed human RCT that specifically analyzed PDRN for the prevention of post-procedural scarring, enrolling 44 patients undergoing open thyroidectomy, who were randomly assigned to receive either intradermal injections of PDRN on days one and two, or no solution [27]. At the three-month follow-up of participants in the study by Kim et al., it was seen that the PDRN-treated group had a significantly lower vascularity subscore on the modified Vancouver Scar Scale (mVSS) (0.476 ± 0.512 versus 0.900 ± 0.447, p = 0.010), a significantly lower erythema index measured by objective colorimetry, and a significantly lower scar height than controls. The total mVSS score tended toward improvement (1.619 + 1.244 versus 2.500 + 1.540; p = 0.059), which did not meet the traditional significance level, most likely due to the small sample size rather than a true effect. A statistically significant reduction in vascularity, which is the subscale most reminiscent of the post-procedural erythema, was obtained with only two injections in the immediate postoperative setting, a clinically feasible protocol. This trial was the first RCT that showed that post-procedural erythema in human subjects can be reduced quantitatively by peri-procedural PDRN administration, thus directly supporting the use of this technique in the post-aesthetic procedure setting [27].

PDRN in aesthetic injection therapies and biostimulation

PDRN and its larger molecular weight analog, PN, have also been designed as injectable meso-aesthetic agents in their own right, albeit as dermal fillers and as biostimulators. One such commercial product is Rejuran (PharmaResearch Co., Gangneung-si, Gangwon, South Korea), a purified 20 mg/mL PN injectable, which has found widespread use in the Asian aesthetic market [35,36], and Nucleofill (Promoitalia, Milan, Italy), a PN-based medical device that has received approval in several countries in Europe [37]. The aesthetic uses of these products include rejuvenating the skin of the face, including the reduction of wrinkles, periocular areas, and bioremodeling. These uses are not identical but are complementary to their application in post-procedural recovery.

The split-face RCT by Lee et al., which included 27 subjects who were injected with PN filler into their periocular area on one side and non-crosslinked hyaluronic acid (HA) filler on the other side, was a methodologically sound comparative evidence of injectable PN/PDRN in an aesthetic indication [38]. The primary endpoint analysis showed no statistically significant difference between PN and HA in Visual Analogue Scale (VAS) or Global Aesthetic Improvement Scale (GAIS) measurements, thereby demonstrating that PN has short-term aesthetic effects comparable to an established standard of care. Nevertheless, the stabilization of the improvement in skin elasticity and hydration was greater with PN than with HA after 28 weeks of observation. This difference has pragmatic value: HA fillers result in short-lived yet volumizing effects, which fade due to degradation by hyaluronidase [39], whereas PN/PDRN lead to structural changes in the ECM by activating fibroblasts [40]. In patients whose procedures cause a loss of ECM integrity (such as laser resurfacing or deep chemical peels), the biostimulatory effect of PDRN could thus have long-term benefits that extend beyond immediate post-procedural recovery.

Lee et al. conducted a prospective comparative study in 40 participants with female pattern hair loss (FPHL) over 12 weeks, evaluating the efficacy of intradermal PDRN monotherapy versus PDRN-plus-PRP combination [41]. Hair count improved in both groups; however, the PDRN-plus-PRP combination demonstrated higher efficacy than PDRN monotherapy alone in improving hair thickness. This outcome was likely attributed to the scalp tissue microenvironment, which recapitulates the pathophysiology of post-procedural skin, characterized by an inflammatory and healing milieu [42]. The proposed mechanism is supported by the complementary and potentially synergistic effects of the A2AR-mediated angiogenic stimulation of PDRN [43] and the growth factor-rich bioactive environment provided by PRP. This principle of combination efficacy can be directly translated into the context of post-procedural treatments that involve the combination of PDRN and PRP, which is increasingly observed in clinical practices but has yet to be tested in controlled trials of aesthetic procedures [44].

PDRN formulations, routes of delivery, and practical considerations

Clinical literature considers a wide variety of routes of PDRN administration, including intramuscular, subcutaneous, perilesional, intradermal, topical, and ophthalmic, which may have different pharmacokinetic implications. The most frequent application of PDRN, both intradermally and in the aesthetic post-procedural context, is directly delivered to the dermal fibroblast-rich milieu where the effects of A2AR are most relevant. The intradermal route offers localized high concentrations at the target tissue and limits systemic exposure, which is consistent with the desirable adverse-event profile across all clinical studies. Topical PDRN application can also be relevant during the post-ablative period (and the frequent absence of the epidermal barrier) when percutaneous penetration of macromolecules is significantly increased [45,46].

Formulation properties, particularly molecular weight, played a key role in determining the pharmacological activity of PDRN; however, these properties were not consistently or fully reported in the published literature. Hwang et al. showed that middle-range molecular weight PDRN (50-1,500 kDa) led to increased collagen composition in wound healing, although apparent surface healing was not significantly different across molecular sizes [21]. There is a range of commercial PDRN preparations varying in the distribution of molecular weights, concentration, and the addition of adjuvant components, including HA, and it is difficult to draw a direct comparison between the products based on the available clinical information. This heterogeneity of formulation is a threat to the utility of the existing evidence base, and standardization is a key concern for future research [21].

Clinicians should be aware of the important distinction between PDRN and its higher molecular weight structural analog, PN. The same fundamental mechanism of A2AR agonism and activation of the salvage pathway exists in PDRN and PN, which differ only in the length of the polymer chains (which is higher in PN), imparting viscoelasticity and structural filler properties in addition to biological activity [47]. The terms are inconsistently used in clinical practice, and the distinction is not always reliably found in the published literature. In the application of post-procedural recovery, in which the primary objective is biological modulation of the healing environment and not volumization, the pharmacological processes involved in A2AR agonist and salvage pathway activity overlap between PDRN and PN; therefore, PN-related studies were considered mechanistically translatable to PDRN in the context of post-procedural recovery reviewed herein, given their shared A2AR agonism and salvage pathway activity [47].

Evidence synthesis: what the current literature supports and what it does not

What the Evidence Supports

Combined, the evidence in place offers solid arguments in favor of three basic conclusions. First, PDRN reliably increases wound re-epithelialization in experimental human trials. PDRN can have statistically significant effects, such as a percentage increase in re-epithelialization after one week or a decrease in overall wound healing time of almost half compared to standard care. Second, peri-procedural administration of PDRN shows a significant decrease in objective indicators of post-procedural erythema and vascularity, as proven in the only study to test it. Third, PDRN prevents melanogenesis via MITF and tyrosinase signals, which offers a mechanistic explanation of PIH prevention that, although yet to be demonstrated in a powered clinical trial, is supported by in vitro data and a consistent cellular paradigm.

The safety data from published trials are uniformly encouraging [12]. Numerous patient exposures in the existing clinical literature [12] have not reported any serious patient adverse events attributable to PDRN. Any events observed were minor and self-limiting [48]. This desirable profile is mechanistically inferred from the deproteinized manufacturing process that removes immunogenic epitopes and from PDRN's biological relationship with endogenous nucleotides [12,49].

What is Not Yet Supported by the Evidence?

Despite this promising outlook, the limitations in the evidence base prevent the development of certain treatment guidelines. Most importantly, no published human RCT has compared PDRN as a post-procedural modifier in the setting of a laser, chemical peel, microneedle, or radiofrequency treatment in an aesthetic patient group, specifically. The available evidence for fractional CO₂ laser in this context remained solely preclinical, with no published human RCT evaluating its use as a post-procedural modifier in an aesthetic patient group [32]. While mechanistically credible, the PRK RCT of the cornea was performed within an ophthalmic environment with an entirely different tissue architecture, limiting its direct translatability to aesthetic post-procedural skin recovery [33]. Studies involving surgical wounds were the best designed and carried out in older and usually medically complex patients with chronic wounds, and not in the immunocompetent, younger to middle-aged aesthetic populations [23,27,31]. Moreover, even the optimal PDRN regimen for aesthetic post-procedural recovery, including optimum concentration, molecular weight, volume, depth of injection, time after surgery, and number of sessions, has not yet been determined by clinical evidence. The trials found in this review used dosing regimens suitable for wound care indications (mostly the 5.625 mg intramuscular formulation approved in Italy), rather than the intradermal microbolus technique normally utilized in aesthetics [12,23,24,47]. This type of dose-finding/protocol optimization work requires a systematic pursuit to extrapolate these regimens to current aesthetic clinical practice. The post-procedural literature has virtually no information on long-term follow-up data beyond three months, making it impossible to describe PDRN's longevity as a healing agent or to discover any delayed adverse events.

Future research directions

The research areas that should be definitely and significantly addressed in the future are clear. The most urgent requirement is appropriately powered, procedure-specific, double-blind RCTs in human aesthetic cohorts. An alternative pragmatic Phase II/III study would include patients receiving standardized fractional ablative CO₂ laser treatment, with a randomized comparison of intradermal PDRN injections versus controls (undergoing the procedures under study). Primary endpoints would include the duration of erythema, assessed using validated colorimetry, and the time to full re-epithelialization, assessed using trans-epidermal water loss. Standardized photography evaluation by blinded judges, patient outcome on a validated post-intervention downtime scale, PIH incidence at 12 weeks, and six-month scar quality are some of the secondary endpoints. Using the magnitude of effects found in the literature on surgical wounds [22,23,24,27,29,31], calculations indicate that a sample size of 80-120 participants will have 80% power to find a clinically meaningful reduction rate of the erythema period, based on conservative estimates of the effect.

Parallel dose-optimization trials are also required to establish the lowest effective PDRN dosage, the pharmacokinetically optimal molecular weight range to be used intradermally in aesthetics, and the interaction between the timing of treatments and clinical response. Whether pre-procedural PDRN priming (i.e., given one to two weeks prior to the aesthetic procedure) contributes to post-procedural effects is also a clinically feasible and biologically plausible hypothesis, warranting formal testing. Relative to PDRN versus PRP, growth factor preparations and low-level laser therapy would set the relative clinical and pharmacoeconomic worth of PDRN as a post-procedural reagent. Lastly, research involving a heterogeneous population across Fitzpatrick skin types is critical, especially if the anti-melanogenic properties of PDRN are to be studied as a PIH prevention agent. This potential use of PDRN in aesthetic dermatology worldwide is arguably the most clinically influential, where darker populations represent a large and swiftly increasing patient demographic.

## Conclusions

PDRN is a biologically well-defined regenerative agent whose dual pharmacological action (anti-inflammatory, angiogenic, and anti-melanogenic effects mediated by the A2AR) is combined with salvage-pathway-mediated cellular proliferation. PDRN has shown reliable effectiveness over placebo and standard care with regard to wound re-epithelialization, erythema, and scar prevention in controlled clinical trials and demonstrates a standard, favourable adverse-event profile. Relative evidence in comparison to active controls indicates comparable effects in short-term results and potentially high biologically mediated durability, in contrast to hyaluronic acid. Preclinical studies have proven its usefulness through fractional ablative CO₂ laser treatment due to its direct signaling on the well-established processes of VEGF upregulation, greater granulation tissue formation, and microvessel neogenesis. Nevertheless, the existing evidence base, though mechanistically coherent and clinically promising, is not sufficient to drive conclusive practice recommendations.

The lack of available procedure-specific human RCTs in aesthetic groups, small participant groups, heterogeneity of the formulation, open-label studies, and limited durations of follow-up in the literature requires the critical distinction of biological plausibility versus clinical evidence. The use of PDRN in post-procedural settings by clinicians today is based on a firm mechanistic rationale and even systematic evidence of the translational kind, yet in a context of informed clinical utilization rather than guideline-based recommendation. The discipline is at an inflection point: the biological explanation is robust enough, and the safety data are safe enough that the value of properly designed aesthetic RCTs is worth the investment and long overdue. PDRN is one of the most scientifically researched agents for formal investigation in post-procedural aesthetic recovery, and high-quality trials in the future could facilitate its transition into a promising adjunct within an evidence-based standard of care.
